# Changing incidence of hepatitis B and persistent infection risk in adults: a population-based follow-up study from 2011 in China

**DOI:** 10.1186/s12889-023-15130-y

**Published:** 2023-02-06

**Authors:** Xiaolan Xu, Chensi Wu, Zhuoqi Lou, Chunting Peng, Lushun Jiang, Tianxian Wu, Taiwen Zeng, Yin Dong, Bing Ruan

**Affiliations:** 1 State Key Laboratory for Diagnosis and Treatment of Infectious Diseases, National Clinical Research Center for Infectious Diseases, National Medical Center for Infectious Diseases, Collaborative Innovation Center for Diagnosis and Treatment of Infectious Diseases, The First Affiliated Hospital, Zhejiang University School of Medicine, 310000 Hangzhou, China; 2 Zhejiang Provincial Peoples’s Hospital, 310000 Hangzhou, China; 3 People’s Hospital Medical Community of Yuhuan County, 318000 Taizhou, China

**Keywords:** Asymptomatic infections, Carrier State, Epidemiology, Hepatitis B virus, Immunization

## Abstract

**Background:**

This study aimed to estimate hepatitis B incidence and chronicity risk in rural adults in China under the background of eliminating viral hepatitis.

**Methods:**

Hepatitis B surface antigen (HBsAg) screening was conducted every 2 years in demonstration areas since 2011. Individuals with baseline HBsAg-negative were included. Incidence was calculated as the number of HBsAg-positive cases divided by the total person-times. HBsAg-positive individuals were followed up to study the persistent infection (> 6 months), chronic infection (> 12 months), and recovery with hepatitis B surface antibody (anti-HBs). The chi-square test and cox proportional regression analysis were performed.

**Results:**

There were 8,942 incident cases over 2,138,532 person-years, yielding an average incidence of 0.42 per 100 person-years. HBV incidence decreased rapidly in both genders and all age groups and then kept stable. Male gender, low population density, low gross domestic product per capita, and islanders were associated with higher incidence. Of the positive cases, 4,989 (55.8%) patients were followed up. The persistent infection, chronic infection, and recovery with anti-HBs rates were 32.3%, 31.0%, and 31.4%, respectively. Persistent or chronic infection was more common in younger adults and males, while seroconversion had no concern with gender or age.

**Conclusions:**

HBV incidence in adult rural residents was decreasing and stayed low. The chronicity rate was relatively high and protective antibodies were induced in only one third. The importance of population-based screening and vaccination for susceptible individuals should be addressed.

**Supplementary Information:**

The online version contains supplementary material available at 10.1186/s12889-023-15130-y.

## Introduction

In 2015, 257 million population were living with hepatitis B virus (HBV) in the world and one-third were living in China, causing nearly 900 thousand deaths yearly [1, 2]. Thanks to the policy of HBV vaccination for newborns, HBV prevalence in people aged 1–59 declined from 9.8 to 7.2% between 1992 and 2006, and it decreased from 9.7 to 1.0% in children under the age of 5, according to the nationwide epidemiology surveys [3, 4]. Based on model predictions, there would still be 60 million cases of HBV infection and 680,000 deaths caused by the disease in China in 2030 [5]. Research on HBV incident cases helps reveal the current disease burden and guides the formulation of targeted prevention and control policies. The national surveillance data revealed that the reporting incidence has decreased to 68.7/100 000 in 2016 in China [6]. Current cohort studies mainly focus on HIV-positive individuals, blood donors, and men who have sex with men [7, 8], thus, large-scale cohort studies in the general population are urgently needed.

Chronic infection is defined by the presence of hepatitis B surface antigen (HBsAg) for at least 6 months [9]. However, recent studies recommended that the time to determine chronic infection should be prolonged [10, 11]. Ito et al. revealed that 19% of patients exhibited prolonged cases of acute infection, in which HBsAg would be cleared between 7 and 12 months [10]. According to previous studies, the chronicity rate was less than 10% in acute infection acquired in adulthood [12]. But the study population were mainly patients admitted to hospital with a small sample size. A cohort study involving 563 patients with clinical symptoms demonstrated that acute HBV infection was self-limited in 96.8% of adults [13]. HBsAg clearance was more common in those with clinical symptoms, abnormal laboratory tests, or fulminant hepatitis, while 70% of the acutely infected adults were asymptomatic [10, 14]. Research on acute HBV infection and its prognosis in asymptomatic patients was seldom conducted. It was reported in a village in Alaska that chronic carriers after acute infection were significantly younger than non-carriers, and 10.4% (5/48) of the general population infected at the age of 20 years or older became chronic carriers [15]. More than 90% of patients hospitalized for acute viral hepatitis B would develop hepatitis B surface antibody (anti-HBs) [13]. Similarly, additional research was required to validate the emergence of anti-HBs after acute infection in general population (mostly asymptomatic).

**Fig. 1:**
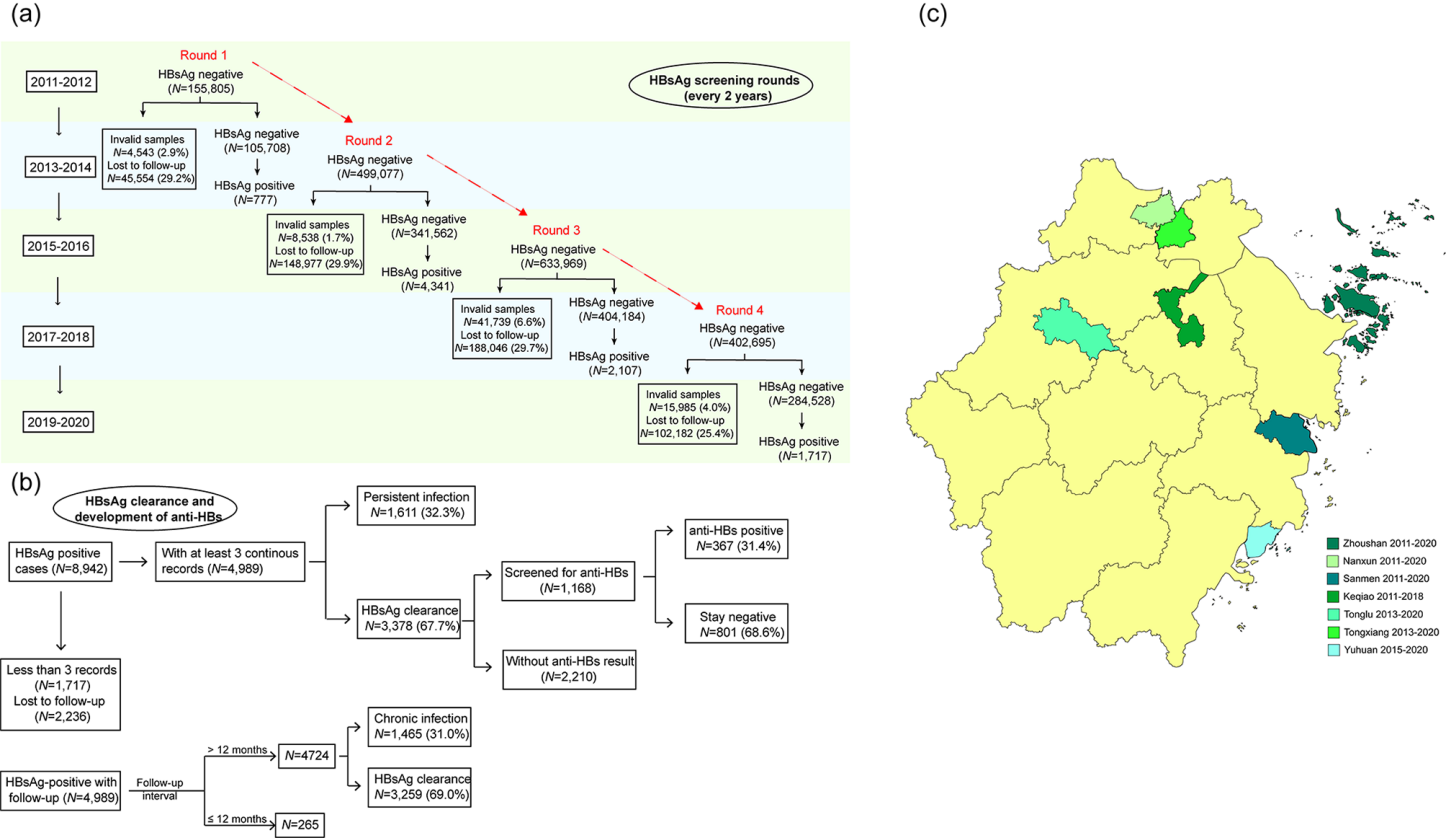
Simplified chart of hepatitis B screening rounds (a), outcomes of incident HBsAg positive cases (b), and the investigated districts distribution in Zhejiang Province (c). Different districts and counties had different periods to do the screening as illustrated on the map. HBsAg, hepatitis B surface antigen; anti-HBs, hepatitis B surface antibody

To meet the WHO’s goal of reducing viral hepatitis incidence by 90% by 2030 [1], the “finding all susceptible persons and infections, vaccinating susceptible persons, following-up infections and treating patients” strategy was presented by the Mega-projects of Science Research for the 11th, 12th, and 13th Five-Year Plan of China. In order to balance the regional macro-level characteristics (geographical location, population density, economic development, HBV prevalence) for comparison, Keqiao district, Nanxun district, Sanmen county, Tonglu county, Tongxiang city, Yuhuan city, and Zhoushan city in Zhejiang province were selected as demonstration areas (Fig. 1, Table S1). Our study aims to estimate the dynamic change of HBV incidence and the persistent infection risk in rural Zhejiang Province.

## Methods

### Study population

The follow-up study was conducted between 2011 and 2020. Rural residents living there for at least 6 months at the time the study was conducted were screened for HBsAg every 2 years, in addition some were screened for anti-HBs additionally. Residents aged 15–100 years who have participated in at least two consecutive screening with baseline HBsAg negative were included. Two consecutive screenings were regarded one round. Four different rounds of 2011.01–2014.12 (Round 1), 2013.01–2016.12 (Round 2), 2015.01–2018.12 (Round 3), and 2017.01–2020.12 (Round 4), each with baseline and follow-up, were analyzed (Fig. 1). The screening was carried out every 2 years in Nanxun, Sanmen, and Tongxiang between 2011 and 2020. It was initiated in 2013 in Tonglu and Tongxiang and in 2015 in Yuhuan. The screening was completed every 2 years from 2011 to 2018 in Keqiao (not in 2020 because of the sudden outbreak of COVID-19).

### Quality control

The standard operation procedure and quality control has been described previously [16, 17]. In brief, the expert group formulated a unified implementation plan and operating procedures to guide project design, epidemiological investigations, laboratory inspections, field training, and data analysis. Field investigators were trained in grades, and a unified laboratory work manual was issued to ensure that blood collection, serum separation, aliquoting, coding, and transportation meet laboratory requirements. All samples were tested with uniform methods.

### Ethical issues

The study was approved by the Human Research Ethics Committee of the First Affiliated Hospital, School of Medicine, Zhejiang University (No. 2017 − 376). Informed consent was obtained from the participants.

### Specimen collection and laboratory testing

We integrated HBsAg and anti-HBs test into the National Free Health Check-up for Residents Program (more details in part 1 of Supplementary Material). We collected 5 ml of venous blood without any anticoagulant and their demographic information from each participant. Samples were stored at a temperature of 0 ~ 4 degrees centigrade and delivered to KingMed Diagnostics Co., Ltd. (Hangzhou, China) for serologic test. The blood serum was tested for HBsAg and anti-HBs by commercially available enzyme immunoassay kits (Wantai Biological Pharmacy Co., Ltd., Beijing, China) and positive samples were confirmed by quantitative kits (Abbott Laboratories, Chicago, IL, USA), as per manufacturers’ instructions. The HBsAg detection range was 0.05 ~ 250 IU/ mL and HBsAg < 0.05 IU/ mL was considered negative. Anti-HBs > 10 IU/L was defined positive.

### HBV incidence and persistent/chronic infection

The primary outcome was HBV incidence calculated as the number of new infections divided by the total person-time in the follow-up. An incident case was identified by the first HBsAg positive result during follow-up. Follow-up time for each patient was the time interval between the date of baseline screening and the date of the second screening, and total person-time was the sum of follow-up time for all participants. We reported the incidence as cases per 100 person-years with 95% confidence intervals (CI). Regional macro-level characteristics such as the population density, gross domestic product (GDP) per capita, HBV prevalence, and geographical location were analyzed for associations with HBV incidence, as these factors indicated the area economic development, source of infection, and hygiene conscious (Table S1). The population density, GDP per capita, and HBV prevalence were collected from the Statistical Yearbook or our previous work and divided into high or low group in the order from highest to lowest (more details in part 2 of supplementary material).

The secondary outcome was the persistent/chronic infection and development of anti-HBs in those who have achieved HBsAg clearance. According to Ito’s suggestion, HBsAg-positive for more than 12 months may be suitable for defining the progression of chronicity [10]. Thus, HBsAg clearance was defined as a loss of HBsAg in less than 12 months. Patients with HBsAg-positive after 6 months and 12 months were considered persistent infection and chronic infection, respectively.

### Statistical analysis

Medians and interquartile ranges (IQR) depicted data that were not normally distributed. The chi-square test was applied for categorical variables. Univariable and multivariable cox proportional regression methods were used to estimate hazard ratios for risk factors of HBV incidence. The stepwise procedure was used for variable selection. Association of gender or age with persistent HBV infection were analyzed by chi-square and multivariable cox proportional regression analysis. P values were two-sided and P < 0.05 indicated that the difference was statistically significant. Statistical analysis was performed by SPSS Statistics 20.0 software (International Business Machines Corporation, Armonk, New York).

## Results

### Study participants characteristics

In total, 155 805, 499 077, 633 969, and 402 695 participants with baseline HBsAg-negative in Round 1–4 were included in our study. During the follow-up, 45 554, 148 977, 188 046, and 102 182 participants did not attend the subsequent screening in Round 1–4 respectively. In addition, 4543, 8538, 41 739, and 15 985 follow-up samples were invalid for testing in Round 1–4 respectively. Finally, we had 105 708, 341 562, 404 184, and 284 528 repeated individuals in each round respectively, included in the analysis (Fig. 1). Compared to participants who had follow-up results, those who were lost to follow-up or with invalid samples were younger at the time of baseline screening and with higher proportion of males (Table S2).

There were 60.3%, 60.6%, 58.1%, and 57.1% female participants in Round 1–4 respectively. The median age at the time of follow-up screening was 61 (IQR 52–68), 63 (IQR 55–69), 65 (IQR 60–71), and 68 (IQR 62–73) years respectively. The proportion of individuals aged under 45 declined from 10.6 to 1.6%, while people aged over 55 increased from 70.3 to 91.0%. The total follow-up time were 188 883, 629 622, 769 964, and 550 064 person-years respectively in 4 rounds (Table 1). The median follow-up time were 1.97 (IQR 1.61–2.01), 1.95 (IQR 1.63–2.05), 1.97 (IQR 1.76–2.10), and 1.98 (IQR 1.69–2.15) years, respectively in each round.

**Table 1:** Baseline demographic characteristics and follow-up time of the repeat participants in the 2-year-round screening

	2011–2014 (Round 1)		2013–2016 (Round 2)		2015–2018 (Round 3)		2017–2020 (Round 4)	
	N (%)	Person-years	N (%)	Person-years	N (%)	Person-years	N (%)	Person-years
Total	105,708(100)	18,8883	341,562(100)	629,622	404,184(100)	769,964	284,528(100)	550,064
Gender								
Male	41,994 (39.7)	74,040	134,460 (39.4)	246,024	169,284 (41.9)	321,875	121,449 (42.7)	233,596
Female	63,714 (60.3)	114,843	207,102 (60.6)	383,598	234,900 (58.1)	448,088	163,079 (57.3)	316,468
Age at the time of follow-up screening (year)								
15–34	2,860 (2.7)	5,187	6,790 (2.0)	13,116	5,121 (1.3)	9,995	1,215 (0.4)	2,443
35–44	8,316 (7.9)	15,174	15,501 (4.5)	30,114	10,598 (2.6)	20,664	3,504 (1.2)	6,969
45–54	20,172 (19.1)	36,809	59,212 (17.3)	116,641	49,291 (12.2)	94,021	21,009 (7.4)	40,417
55–64	34,328 (32.5)	60,859	113,017 (33.1)	213,111	119,214 (29.5)	222,630	68,604 (24.1)	129,782
65–74	26,142 (24.7)	46,647	101,894 (29.8)	176,795	155,268 (38.4)	299,196	132,698 (46.6)	257,168
75+	13,890 (13.1)	24,207	45,148 (13.2)	79,845	64,692 (16.0)	123,458	57,498 (20.2)	113,285
Population density								
High	2,693 (2.5)	4,265	103,017 (30.2)	163,771	141,323 (35.0)	252,830	138,532 (48.7)	275,706
Low	103,015 (97.5)	184,618	238,545 (69.8)	465,851	262,861 (65.0)	517,134	145,996 (51.3)	274,358
Gross domestic product per capita								
High	69,751 (66.0)	135,969	143,204 (41.9)	278,718	188,794 (46.7)	353,494	73,410 (25.8)	141,427
Low	35,957 (34.0)	52,914	198,358 (58.1)	350,904	215,390 (52.3)	416,470	211,118 (74.1)	408,637
Prevalence								
Low	6,644 (6.3)	10,706	35,536 (10.4)	70,684	103,140 (25.5)	170,723	127,152 (44.7)	243,376
High	99,064 (93.7)	178,176	306,026 (89.6)	558,938	301,044 (74.5)	599,241	157,376 (55.3)	306,688
Geographical location								
Non-island area	99,064 (93.7)	178,176	319,518 (93.5)	583,302	333,807 (82.6)	660,575	196,949 (69.2)	382,701
Island area	6,644 (6.3)	10,706	22,044 (6.5)	46,320	70,377 (17.4)	109,388	87,579 (30.8)	167,363

High population density: Tongxiang, Yuhuan, and Zhoushan; high gross domestic product per capita: Keqiao, Zhoushan, and Yuhuan; high HBV prevalence: Yuhuan, Zhoushan, Tonglu, and Sanmen; island areas: Zhoushan, Yuhuan, and Sanmen

### Average incidence and risk factors

We observed 8,942 new HBV-infected cases in total with an overall follow-up time of 2,138,532 person-years, yielding an average HBV incidence of 0.42 per 100 person-years (95% CI: 0.41–0.43) in rural residents in recent 10 years.

It was only during the 2015–2018 screening round that all seven demonstration areas completed HBsAg screening among rural residents, with the most participants. Thus, we conducted a risk factor analysis with the data in Round 2015–2018. The assumption of proportional hazards was tested (Figure S1). Gender, population density, GDP per capita, HBV prevalence, and geographical location were associated with HBV incidence in univariate analysis (Table 2). Geographical location was the most influential factor in the multivariate regression analysis. Incidence in islanders (people living in Zhoushan, Yuhuan, and Sanmen) was 3.80 times that of non-islanders (95%CI: 3.13–4.61, P < 0.001). Lower GDP per capita (hazard ratio: 1.76, 95% CI: 1.58–1.95, P < 0.001) and males (hazard ratio: 1.15, 95% CI: 1.06–1.26, P = 0.001) were associated with higher HBV incidence. High population density was a risk factor in univariate analysis (hazard ratio: 1.30, 95% CI: 1.19–1.42, P < 0.001) but showed a lower risk for infection compared with low population density in multivariate analysis (hazard ratio: 0.80, 95% CI:0.73–0.89, P < 0.001). Additionally, there was no association between HBV prevalence and incidence after adjusting for multiple variates. Similarly, age was not associated with HBV incidence in both univariate and multivariate analysis.

**Table 2:** HBV incidence per 100 person-years and risk factors in Round 2015–2018

Characteristics	Cases/Person-years	Incidence (95%CI)	Univariate analysis		Multivariate analysis	
			Hazard ratio (95%CI)	P-value	Hazard ratio (95%CI)	P-value
Total	2,107/769,964	0.27 (0.26–0.29)				
Gender						
Female	1,169/448,088	0.26 (0.25–0.28)	Ref		Ref	
Male	938/321,875	0.29 (0.27–0.31)	1.11 (1.02–1.21)	P = 0.018	1.15 (1.06–1.26)	P = 0.001
Age at the time of follow-up screening (year)						
15–34	22/9,995	0.22 (0.14–0.33)	Ref		Ref	
35–44	73/20,664	0.35 (0.28–0.44)	1.58 (0.98–2.54)	P = 0.062	1.37 (0.85–2.20)	P = 0.201
45–54	294/94,021	0.31 (0.28–0.35)	1.40 (0.91–2.16)	P = 0.126	1.30 (0.84–2.00)	P = 0.244
55–64	625/222,630	0.28 (0.26–0.30)	1.25 (0.82–1.92)	P = 0.299	1.19 (0.78–1.82)	P = 0.423
65–74	790/299,196	0.26 (0.25–0.28)	1.12 (0.73–1.71)	P = 0.611	1.16 (0.75–1.77)	P = 0.509
75+	303/123,458	0.25 (0.22–0.27)	1.02 (0.66–1.57)	P = 0.930	1.01 (0.65–1.57)	P = 0.958
Baseline population density						
Low	1,316/517,134	0.25 (0.24–0.27)	Ref		Ref	
High	791/252,830	0.31 (0.29–0.34)	1.30 (1.19–1.42)	P < 0.001	0.80 (0.73–0.89)	P < 0.001
Baseline gross domestic product per capita						
High	822/353,494	0.23 (0.22–0.25)	Ref		Ref	
Low	1,285/416,470	0.31 (0.29–0.33)	1.11 (1.02–1.21)	P = 0.023	1.76 (1.58–1.95)	P < 0.001
Baseline prevalence						
Low	1,329/599,241	0.22 (0.21–0.23)	Ref		Ref	
High	778/170,723	0.46 (0.42–0.49)	2.39 (2.19–2.62)	P < 0.001	1.13 (0.97–1.32)	P = 0.116
Geographical location						
Non-island area	1,525/660,575	0.23 (0.22–0.24)	Ref		Ref	
Island area	582/109,388	0.53 (0.49–0.58)	2.92 (2.66–3.22)	P < 0.001	3.80 (3.13–4.61)	P < 0.001

High population density: Tongxiang, Yuhuan, and Zhoushan; high gross domestic product per capita: Keqiao, Zhoushan, and Yuhuan; high HBV prevalence: Yuhuan, Zhoushan, Tonglu, and Sanmen; island areas: Zhoushan, Yuhuan, and Sanmen

### Dynamic changing HBV incidence in each cohort

**Fig. 2:**
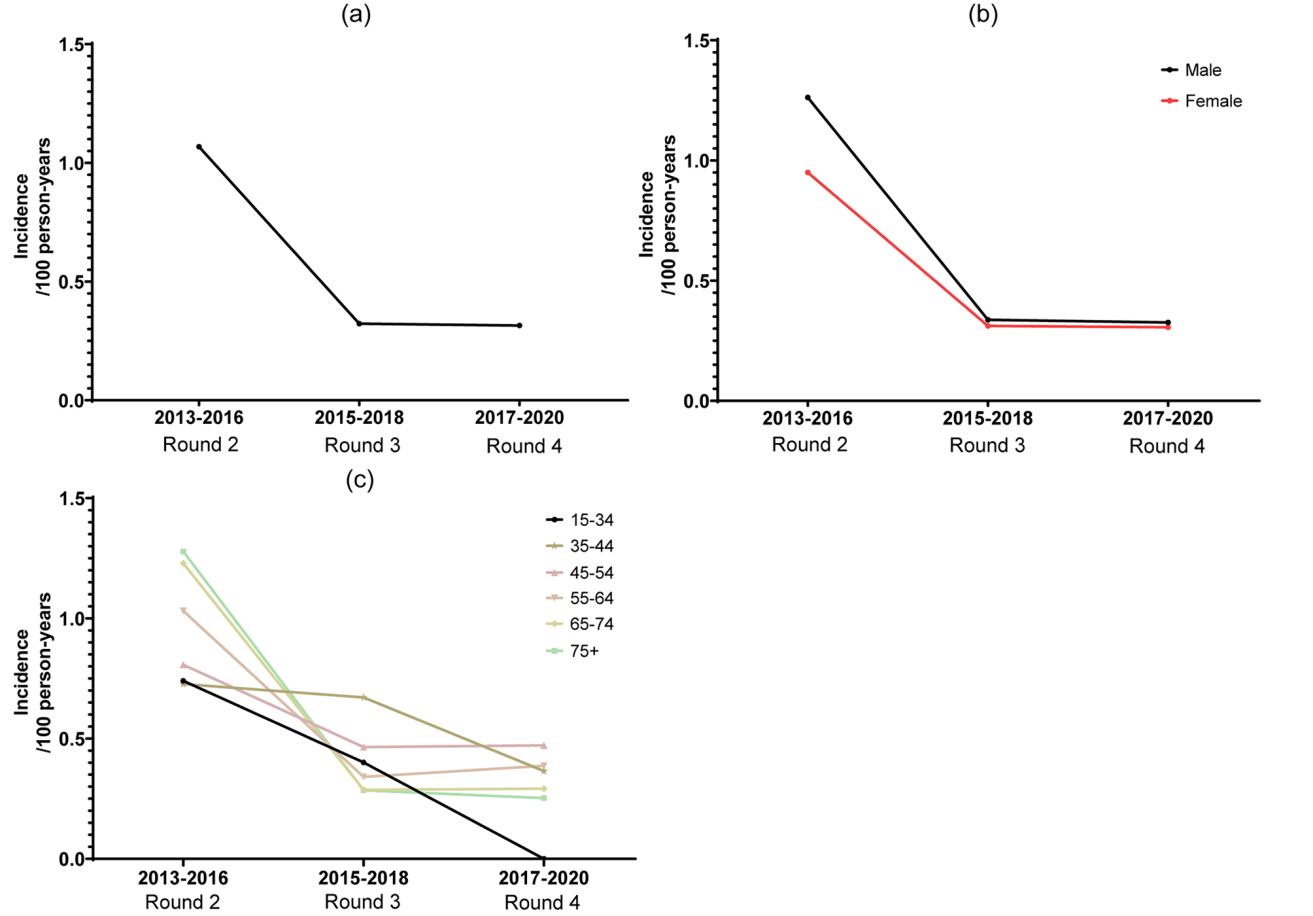
Changing incidence in regions that have carried out hepatitis B screening between 2013 and 2020 (a) Total incidence; (b) Incidence in different genders; (c) Incidence in different age groups

We analyzed the dynamic change of HBV incidence in demonstration areas that had carried out HBV screening between 2013 and 2020 because 2 areas with a substantial number of participants did not initiate screening until 2013. We found a dramatic decline of the HBV incidence (Fig. 2a). The incidence in Round 2, Round 3, and Round 4 was 1.07 (95% CI: 1.04–1.10), 0.32 (95% CI: 0.31–0.34), and 0.31 (95% CI: 0.30–0.33) per 100 person-years, respectively. Compared to Round 2, the incidence in Round 4 was reduced by 78%. HBV incidence in the male gender was higher (1.26, 95% CI: 1.20–1.32 vs. 0.95, 95% CI: 0.91–0.99 per 100 person-years, P < 0.001) initially, so declined more steeply to Round 3 then stabilized, a similar pattern to that in females (0.33, 95% CI: 0.30–0.35 vs. 0.31, 95% CI: 0.28–0.33 per 100 person-years, P = 0.232) (Fig. 2b). In all age groups, incidence showed a decreasing trend (Fig. 2c). There reported no incident HBV infection cases in the 15–34 years age group in Round 4. However, the decline in individuals aged 35–54 years was slow. Incidence in people aged over 65 years was the highest in Round 2 but decreased rapidly, which was the lowest among all age groups in Round 3.

### The persistent infection risk and risk factors

In 8,942 participants who presented a positive HBsAg, 4,989 cases were followed up (2,236 lost to follow-up and 1,717 turned positive in Round 4). There was no difference between participants who completed or lost their follow-up in gender (male ratio: 45.5% versus 44.4%, P = 0.355). However, those who completed the follow-up were older (65 [IQR 60–71] years versus 61 [IQR 51–69] years, P < 0.001). In total, 3,378 individuals presented negative HBsAg results, and 1,611 stayed positive within a median follow-up time of 1.97 years (IQR 1.74–2.16), yielding an HBsAg clearance rate of 67.7% (95% CI: 66.4–69.0%) and a persistent infection rate of 32.3% (95% CI: 31.0–33.6%).

**Fig. 3:**
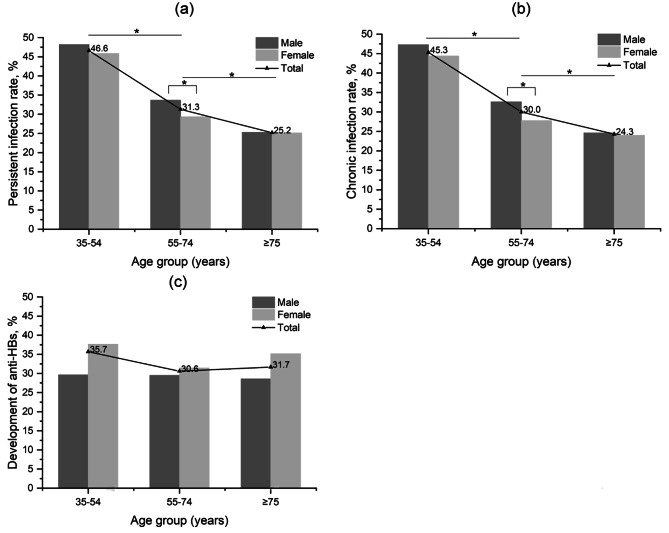
The proportion of persistent infection (a), chronic infection (b), and development of anti-HBs (c) following acute infection in adults in different age groups and genders. The sample size of the 15–34 years age group is too small and is not presented in the figures. Anti-HBs, hepatitis B surface antibody. * P < 0.05 and p-values were two-sided

Overall women and men presented similar persistent infection risk in univariate analysis (31.2% vs. 33.7%, P = 0.064, Table 3), when stratified by age and gender, the risk was slightly higher in men aged 55–74 age group (33.7% vs. 29.3%, P = 0.044, Fig. 3a). Men were marginally associated with a higher risk in multivariate analysis (hazard ratio: 1.14, 95% CI: 1.04–1.26, P = 0.008). The persistent infection rate ranged from 25.2 to 46.6% in various age groups (P < 0.05), showing a decreasing trend from young to elder people (Fig. 3a). People aged 35–54 years still had a higher risk of persistent infection than those aged over 75 after adjusting for gender (hazard ratio: 1.58, 95% CI: 1.32–1.91, P < 0.001).

**Table 3:** Risk factors associated with persistent HBsAg positive

	Total (%)	Persistent infection (95%CI), %	P-value	Multivariate analysis	
				Hazard ratio (95%CI)	P-value
Total	4,989 (100)				
Gender					
Female	2,776 (56.0)	31.2 (29.5–33.0)		Ref	
Male	2,213 (44.0)	33.7 (31.7–35.7)	P = 0.064	1.14 (1.04–1.26)	P = 0.008
Baseline age (year)^a^					
75+	754 (15.1)	25.2 (22.1–28.5)		Ref	
15–34	18 (0.4)	44.4 (21.5–69.2)		1.36(0.67–2.75)	P = 0.398
35–54	605 (12.1)	46.6 (42.6–50.7)		1.58 (1.32–1.91)	P < 0.001
55–74	3,612 (72.4)	31.3 (29.8–32.9)	P < 0.001	1.12 (0.96–1.31)	P = 0.149

^a^The baseline age is calculated with the time at which HBsAg positive was first found and date of birth. HBsAg, hepatitis B virus surface antigen

### The chronicity risk and risk factors

For the subgroup with a follow-up interval > 12 months (N = 4,724), 1,465 patients stayed positive within a median follow-up time of 1.99 years (IQR 1.80–2.18), indicating a chronic infection rate of 31.0% (95% CI: 29.7–32.4%). Men presented higher chronic infection risk both in univariate analysis (33.7% vs. 29.6%, P = 0.023) and multivariate analysis (hazard ratio: 1.18, 95% CI: 1.07–1.31, P < 0.001) (Table 4). The chronicity rate ranged from 24.3 to 45.3% in various age groups (P < 0.05), showing a decreasing trend from young to elder people (Fig. 3b). Similarly, the risk was slightly higher in the male gender among the 55–74 age group (32.6% vs. 27.8%, P = 0.002) but no gender difference in other groups. People aged 35–54 years still had a higher risk of being chronic carriers than those aged over 75 after adjusting for gender (hazard ratio: 1.56, 95% CI: 1.29–1.90, P < 0.001).

**Table 4:** Risk factors associated with chronic hepatitis B infection

	Total (%)	Chronic infection (95%CI), %	P-value	Multivariate analysis	
				Hazard ratio (95%CI)	P-value
Total	4,724 (100)				
Gender					
Female	2,618 (55.4)	29.6 (27.9–31.4)		Ref	
Male	2,106 (44.6)	33.7 (30.7–34.8)	P = 0.023	1.18 (1.07–1.31)	P = 0.001
Baseline age (year)^a^					
75+	712 (15.1)	24.3 (21.2–27.6)		Ref	
15–34	16 (0.3)	37.5(15.2–64.6)		1.09 (0.48–2.46)	P = 0.835
35–54	567 (12.0)	45.3 (41.2–49.5)		1.56 (1.29–1.90)	P < 0.001
55–74	3,429(72.6)	30.0 (28.5–31.6)	P < 0.001	1.10 (0.94–1.30)	P = 0.233

^a^The baseline age is calculated with the time at which HBsAg positive was first found and date of birth. HBsAg, hepatitis B surface antigen

### The development of anti-HBs in HBsAg-clearance patients

We found that 1,168 of those who have achieved HBsAg clearance had available anti-HBs tests. Anti-HBs was detected in 367 participants, yielding a recovery with anti-HBs rate of 31.4% (95% CI: 28.8–34.2%). No difference was found between different genders or age groups in terms of recovery with anti-HBs (Fig. 3c).

## Discussion

Our study demonstrated an average HBV incidence rate of 0.42 per 100 person-years in rural adults during 2011 and 2020. It was higher than what has been reported in the recent study with the national surveillance data [6]. The reported HBV incidence in China has dropped from 88.8 to 100,000 to 68.7 per 100,000 from 2009 to 2016, with an average annual percentage rate of -0.2% [6]. However, only patients who were treated in the hospital were reported in the national system and missing or duplicate reporting was common, which may result in an underestimation [18, 19]. HBV incidence has shown a declining tendency due to the universal vaccination of newborns and the enhancement of patient detection rate and treatment rate. For example, the incidence in US blood donors dropped from 3.4 to 2.4 per 100 000 person-years from 2008 to 2019, with a reduction of 29% [7, 20]. Laboratory surveillance data in England indicated an incidence of 7.4 per 100 000 person-years during 1995 to 2000 [21], and declined to 1.1 per 100,000 person-years in 2008, with a reduction of 85% [22]. Different from the above low-prevalence countries, in higher prevalent areas like Uganda, the incidence reported in a cohort study was as high as 10.5/100 person-years between 2013 and 2015, indicating a heavy prevention burden [23].

HBV incidence in Round 2015–2018 has reduced by 78% compared to that in Round 2013–2016 in our study. Undiagnosed infections were detected by screening and well managed, thus the sources of infection were well controlled. Given the universal vaccination in China became mandatory in 1992, most participants in our study were not vaccinated in their childhood. Free adult immunization for HBsAg-negative individuals were implemented in Zhoushan in 2013 with 53,172 people completing three doses, which was proved effective in lowering the HBV epidemic level [24]. The same vaccination program was also piloted in Keqiao and Tonglu. Our previous economic analysis based on the Markov model has demonstrated that population-based immunization could prevent most new HBV infections if all susceptible individuals were vaccinated and this strategy was cost-effective [25]. However, the interventions may have little impact when HBV incidence has dropped to a lower level and the incidence reached a plateau in the last round.

The decline of HBV incidence in the 15–34 years age group was striking, owing to the planned immunization program for newborns since 1992. Incidence in people aged 35–54 years decreased slightly and stayed high, which could be explained by sexual transmission. Incidence in people aged over 55 years was high in Round 2 but decreased obviously in Round 3, probably because older people were at a low likelihood of exposure to risk factors for HBV infection. In contrast to our study, the national reporting data showed incidence among people over 50 years was increasing [6]. However, as elderly people showed a higher HBV prevalence in previous studies [4], thus they had a greater likelihood to suffer from complications of HBV infection and be treated in the hospital. Besides, older people had a closer contact with hospitals leading to the incidental discovery of HBV infection.

According to our result, men suffered a higher HBV incidence rate which was the same as that reported in Central–Eastern European areas [26]. This may be due to increased exposure to sexual transmission risk factors in men such as homosexual sex and multiple sexual partners [27, 28]. Besides, HBV screening was provided for pregnant women in China thus bringing a higher detection and treatment rate in women. The GDP per capita and population density was negatively associated with HBV incidence. HBV transmission could be reduced in more developed areas because people had more opportunities to receive health education or medical service. It was previously reported that the combination of these two factors played a more important part in HBV transmission than either individually [29]. Higher HBV prevalence in island areas was found in the previous study [30], similarly, our study demonstrated that island areas were also associated with higher HBV incidence. Harborside may associated with higher population mobility. Furthermore, during the fishing season, poorer sanitary conditions on the boat may encourage the transmission of HBV.

The proportion of persistent infection (HBsAg positive > 6 months) and chronic infection (HBsAg positive > 12 months) in adult was 32.3% and 31.0%, respectively, which were specifically higher than that reported previously. Zhang et al. reported a persistent infection rate of 8.5% upon population-based surveillance with 294 acutely infected patients [31]. On one hand, genotype B and C were the most common in China, and genotype C was more prone to chronicity development [31, 32]. Genotype C was more prevalent (> 60%) in patients older than 35 years in Southeast China and the Zhejiang province [33–35]. On the other hand, HBsAg clearance was more often seen in patients with clinical hepatitis, while the new infections detected by screening were often asymptomatic [36, 37]. In the population-based study carried out in remote coastal and river locations of Alaska, during the 4.5-year follow-up, 13.3% of incident cases became chronic carriers, but most of the participants were under 20 years [15]. In cohort studies conducted in symptomatic acute infected hepatitis B patients, the persistent infection risk was 0 in Greece [12], 6.8% in Italy [37], 7.6% in China [38], 12.1% in Germany [39], and 13.4% in Japan [40], respectively. The persistent and chronic infection risk decreased with age. McMahon demonstrated the persistent infection rate was 10.4% and 19.5%, respectively, in people aged over 20 or under 10 years [15]. But few studies involved such a large age span as ours. Men presented a higher risk of persistent infection or becoming chronic carriers than women in individuals aged 55–74 years, which was reported previously [15, 41], while no gender difference was found in other groups.

Anti-HBs incidence in patients who spontaneously cleared HBsAg was disclosed infrequently. Mcmahon et al. reported in 1985 that seropositive of anti-HBs was more likely to appear in older individuals and females, however most participants were under 30 years old, and the sample size was small [15]. Over 90% of acutely infected individuals admitted to the hospital developed anti-HBs within six months, according to Tassopoulos et al. [13], although they did not investigate the effects of age and gender. In our study, only 31.4% of patients generated protective antibodies after HBsAg clearance which was extremely lower than that reported in inpatient or that induced by vaccination [13, 42]. And there was no statistical difference between each age group or gender. This indicated that the prevention and control of HBV infection in high-prevalence areas was still challenging. Screening HBV infection and immunization for young adults have been proved to be cost-effective in China [25, 43]. Free vaccination could be considered for susceptible adults to avoid new infections.

The large percentage of participants who were lost to follow-up may have an impact on our findings given that the lost population was mostly younger males. As was already indicated, both in our study and other research [26], HBV incidence was greater in men, which could lead to an underestimating of HBV incidence due to the lost population. However, in the final two screening rounds, the gender and age differences in HBV incidence were significantly diminished. Younger participants also had a greater likelihood of persistent infection, suggesting that prevention and control were more challenging in this population because it was difficult to monitor their infection situation. Population-based HBV screening should be encouraged considering it was cost-effective in our previous research [25].

Our study has several limitations. First, the long follow-up interval might cause an underestimation of HBV incidence and an overestimation of persistent infection rate because most adults would spontaneously clear HBsAg within 6 months [12]. Second, the long interval and population-based study also caused a high rate of lost to follow-up. It was inevitable because the free check-up program was unified to perform in each demonstration area at a fixed time and site, while some of the participants were not available at the planned time. This could be mitigated by the large sample size in our study. Third, the participants aged 15–34 years were relatively insufficient to analyze the persistent infection risk or recovery with anti-HBs rate in this group. Further, the study was carried out in rural residents living there for at least 6 months and most of them were middle-aged and elderly people. In addition, HBV genotype of the incident cases was not tested, subsequent studies would be conducted to investigate the association between genotypes and persistent infection. Finally, the baseline HBV DNA or biochemical indicators which showed an opposite association with HBsAg clearance in previous studies were not measured so we could not determine their influence on chronicity [10, 37].

## Conclusion

In conclusion, the HBV incidence in rural residents in demonstration areas was decreasing and was particularly lower in high population density areas, high GDP per capita areas, non-island areas, and females. Compared to the 2015 baseline, HBV incidence in 2020 has reduced by 78%. But incidence kept stable in recent years, indicating that the 90% reduction in HBV incidence by 2030 was still challenging. The persistent infection risk was high in adults, especially in males and younger individuals. Adult immunization is of vital importance in eliminating incident HBV infections to fulfill the goal of eliminating viral hepatitis.

## Electronic supplementary material

Below is the link to the electronic supplementary material.

Supplementary Material 1

## Data Availability

The datasets used and analyzed during the current study are available from the corresponding author on reasonable request.

## Abbreviations

HBV: Hepatitis B virus
HBsAg: Hepatitis B surface antigen
anti-HBs: Hepatitis B surface antibody
CI: Confidence intervals
GDP: Gross domestic product
IQR: Interquartile range
