# Supplementary material for: Changing incidence of hepatitis B and persistent infection risk in adults: a population-based follow-up study from 2011 in China
Source: BMC Public Health. 2023 Feb 6;23:256. doi: 10.1186/s12889-023-15130-y (PMC9901124; doi:10.1186/s12889-023-15130-y)
Supplement: Supplementary file 1 — Supplementary Material 1 [file 12889_2023_15130_MOESM1_ESM.docx]

Changing incidence of hepatitis B and persistent infection risk in adults: a population-based follow-up study from 2011 in China

Xiaolan Xu, Chensi Wu, Zhuoqi Lou, Chunting Peng, Lushun Jiang, Tianxian Wu, Taiwen Zeng, Yin Dong, and Bing Ruan

Supplementary Material

1. Introduction of the hepatitis B screening program

Large-scale field epidemiology and intervention study on AIDS, viral hepatitis, and tuberculosis in Zhejiang Province were launched in 2009, under the support of the Mega-projects of Science Research for the 11th, 12th, and 13th Five-Year Plan of China. The strategy of “finding all susceptible persons and infections, vaccinating susceptible persons, following-up infections, and treating patients” was presented in the demonstration areas for the effort to eliminate hepatitis B virus (HBV) infection. To make full use of the existing work base and resources, taking full consideration of the representativeness of the region and infectious disease infection factors, we finally selected Keqiao, Nanxun, Sanmen, Tonglu, Tongxiang, Yuhuan, and Zhoushan as demonstration areas, which covered different landforms and different economic development levels. HBV surface antigen (HBsAg) screening was conducted in the above areas for rural residents aged over 15 years and living there for at least 6 months. HBsAg and HBV surface antibody (anti-HBs) tests were offered free of charge as a supplement to the National Free Health Check-up for Residents Program, which was provided for residents every 2 years for free. Because the National Free Health Check-up for Residents Program was independently organized by the local department of health at a specific time, the HBV screening was not finished simultaneously but discontinuously in the above seven areas. Besides, anti-virus treatment was provided for those who met the therapeutic indication in Sanmen, and immunization was provided for those with HBsAg and anti-HBs negative in Zhoushan, Keqiao, and Tonglu, all for free.

1. Regional macro-level characteristics

The population density and gross domestic product (GDP) per capita were collected from the Statistical Yearbook and divided into high or low group in the order from highest to lowest, respectively.^1-6^ Tongxiang, Yuhuan, and Zhoushan were categorised as areas with high population density. Keqiao, Zhoushan, and Yuhuan were areas with high GDP per capita. Yuhuan, Zhoushan, Tonglu, and Sanmen were areas with high HBV prevalence. Geographical location was categorized as island areas (Zhoushan, Yuhuan, and Sanmen) or non-island areas (Tonglu, Tongxiang, Keqiao, and Nanxun).

Table S1 Sociological, HBV epidemiological, and geographical characteristics of 7 demonstration areas in 2015

|  | Population density (People/km^2^) | | GDP per capita (US$) ^a^ | | HBV prevalence (%) ^b^ | | Geographical location | |
| --- | --- | --- | --- | --- | --- | --- | --- | --- |
| Zhoushan | | 800 | | 18,010 | | 8.90 | | Island |
| Yuhuan | | 857^c^ | | 16,477 | | 9.95 | | Island |
| Tonglu | | 224 | | 13,134 | | 6.95 | | Non-island |
| Sanmen | | 405^c^ | | 6,314 | | 5.62 | | Island |
| Tongxiang | | 948 | | 12,651 | | 4.99 | | Non-island |
| Keqiao | | 613 | | 29,584 | | 5.03 | | Non-island |
| Nanxun | | 696 | | 12,688 | | 4.56 | | Non-island |

^a^ GDP per capita was presented in US$, with an exchange rate of US$1 = 6.2284 China Yuan in 2015.

^b^ HBV prevalence is calculated by the number of positive individuals divided by the total number of participants. Here is the average of the 2015 and 2016 prevalence in our previous work.

^c^ Population density in 2015 was absent in Yuhuan and Sanmen, so we used the data in 2019 instead because population density has been stable for recent 5 years.

GDP, gross domestic product; HBV, hepatitis B virus

1. Additional tables and figures

Table S2 Comparison of age and gender of participants with or without follow-up results in HBsAg-negative participants

|  | with follow-up | without follow-up | *P*-value |
| --- | --- | --- | --- |
| Male, % | | | |
| 2011–2014 (Round 1) | 39.7 | 44.0 | *P* < 0.001 |
| 2013–2016 (Round 2) | 39.4 | 42.5 | *P* < 0.001 |
| 2015–2018 (Round 3) | 40.1 | 41.9 | *P* < 0.001 |
| 2017–2020 (Round 4) | 42.7 | 43.8 | *P* < 0.001 |
| Age (median [interquartile range]), year | | | |
| 2011–2014 (Round 1) | 60 [50**–**67] | 54 [42**–**65] | *P* < 0.001 |
| 2013–2016 (Round 2) | 61 [54**–**68] | 54 [44**–**65] | *P* < 0.001 |
| 2015–2018 (Round 3) | 64 [58**–**70] | 53 [45**–**63] | *P* < 0.001 |
| 2017–2020 (Round 4) | 66 [61**–**71] | 59 [50**–**70] | *P* < 0.001 |

*P*-values were two-sided.


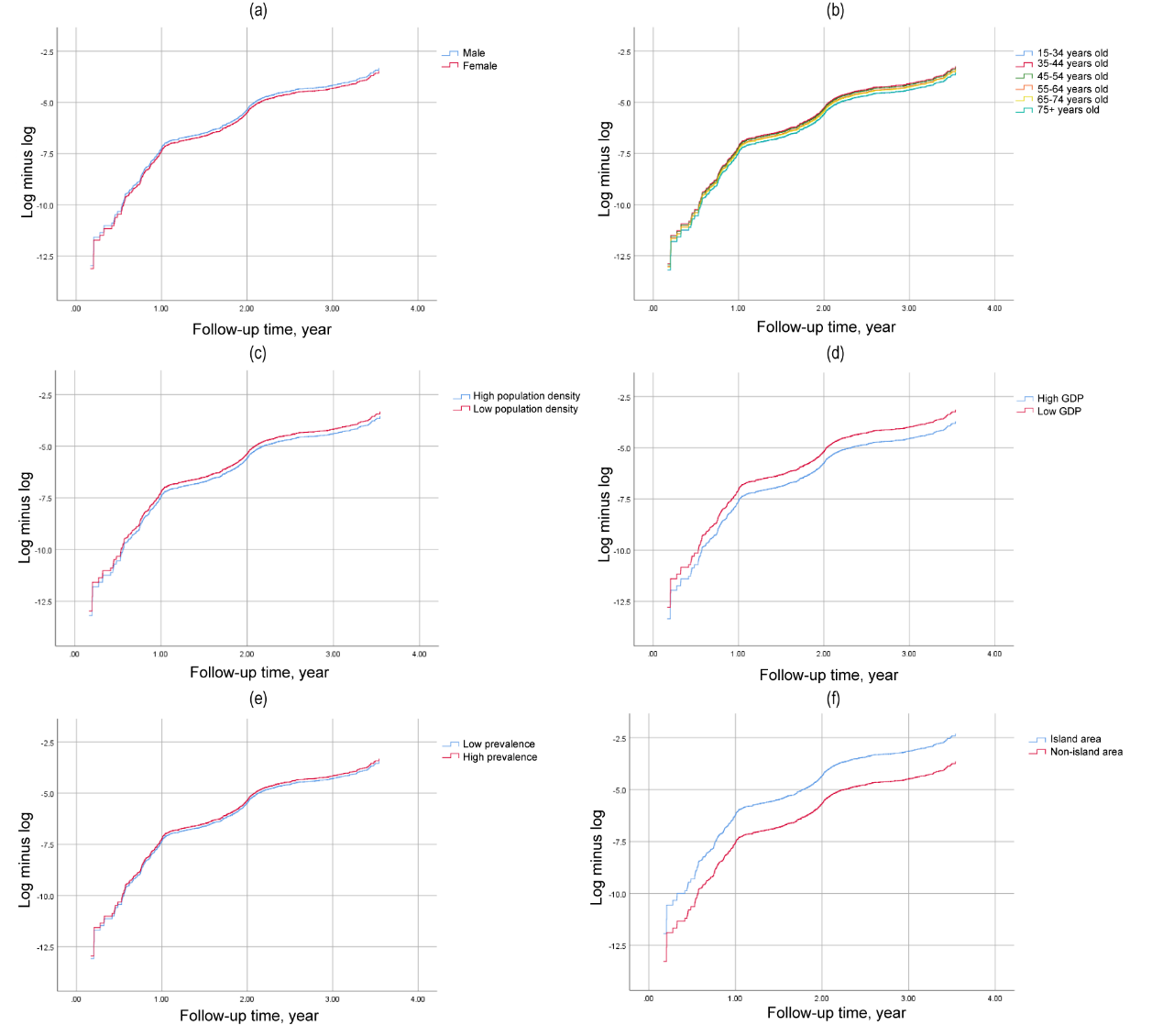


Figure S1 Verification of the equivalent proportional hazards hypothesis of the Cox proportional hazards model (a) Gender; (b) Age group; (c)Population density; (d) Gross domestic product per capita (GDP); (e) HBV prevalence; (f) Geographical location

**Reference:**

1. Statistical Yearbook 2020 of Keqiao District. <http://www.kq.gov.cn/col/col1229451279/index.html>. Updated January 25, 2021. Accessed July 24, 2021.

2. Statistical Yearbook 2020 of Huzhou City. <http://tjj.huzhou.gov.cn/col/col1229208257/>. Updated October 23, 2020. Accessed July 24, 2021.

3. Statistical Yearbook 2020 of Taizhou City. <http://tjj.zjtz.gov.cn/>. Updated December 4, 2020. Accessed July 24, 2021.

4. Statistical Yearbook 2020 of Zhoushan City. <http://zstj.zhoushan.gov.cn/col/col1229471531/index.html>. Updated January 5, 2021. Accessed July 24, 2021.

5. Statistical Yearbook 2020 of Tonglu County. <http://www.tonglu.gov.cn/art/2021/1/15/art_1535189_58961194.html>. Updated January 15, 2021. Accessed July 24, 2021.

6. Statistical Yearbook 2020 of Jiaxing City. <http://tjj.jiaxing.gov.cn/col/col1512382/index.html>. Updated December 31, 2020. Accessed July 24, 2021.
